# Molecular Mechanisms of Hepatotoxicity

**DOI:** 10.3390/ijms24043791

**Published:** 2023-02-14

**Authors:** Antonietta Messina, Jean-Charles Duclos-Vallée

**Affiliations:** UMR_S 1193, INSERM/Université Paris-Saclay, Fédération Hospitalo-Universitaire (FHU) Hépatinov, F-94807 Villejuif, France

Drug-induced liver injury, also known as drug-induced hepatotoxicity (DILI), is a major cause of medicine withdrawal (prescription or over-the-counter) from the market. Caused by a multitude of xenobiotics, among which fungal toxins, dietary supplements and herbal products should not be underestimated, DILI is primarily a clinical manifestation of an unexpected harmful reaction of the liver to drugs, and mimics the etiology and symptoms of many acute and chronic liver diseases [1,2]. With a few exceptions, the mechanisms by which drugs and molecules cause hepatic injury are still elusive; thus, the aim of this Special Issue is to gather the latest findings about the pathways involved in hepatotoxicity, the identification of potential novel targets for the development of new therapeutic strategies, and the development of new devices for improving drug screening and pharmacological responses.

Paracetamol, also called acetaminophen or N-acetyl-p-aminophenol (APAP), is one of the most widely used over-the-counter antipyretic/analgesics. Its toxicity, whether via accidental or intentional overdose, is an ongoing global problem that continues to result in severe cases of hepatotoxicity, acute liver failure, and even irreversible liver injury necessitating liver transplantation. While many studies have confirmed that hepatotoxicity from paracetamol overdose is mainly due to glutathione depletion and the subsequent accumulation of harmful metabolites, new studies, including those by Dong [3] and Gajdošik [4] published in this issue, also highlight the role played by other agents and signaling pathways in APAP-induced liver damage.

Dong and colleagues showed that gp130-dependent Interleukin-11 (IL11) secretion promotes APAP-induced liver damage and inhibits liver regeneration. Using the CRISPR/Cas9 technique, they generated knockout mouse models for Il11 expression (CKO^Il11^) and the gp130 (CKO^gp130^) pathway. Mice from both models, when unable to activate the gp130 signaling pathway or secrete Il11, already showed reduced liver damage (low levels of ALT, AST, and hepatic necrosis) and increased expression of most of the liver regeneration markers after 6 h of treatment with high doses of the drug. Indeed, the authors demonstrated that the secretion of Il11 from APAP-injured hepatocytes, which appear to be the major source of cytokine secretion, probably results in increased cell death through activation of the gp130-mediated-ERK pathway. This, in conjunction with glutathione depletion and accumulation of the toxic metabolite NAPQI, could result in immediate acute liver injury. This is a particularly interesting result given that for many years, the pg130 pathway and Il11 were both considered to have a protective role in mouse models of liver injury; in line with more recent articles [5], the authors showed that aggregate gp130 signaling and Il11 upregulation in a damaged liver are toxic and antiregenerative after APAP treatment [3].

Gajdošik et al., on the other hand, investigated the role of extracellular vesicles (EVs) in APAP-induced hepatoxicity. EVs are release by liver cells, and their morphology and composition depend on the (physio)pathological state of the organ; so, they can be used as markers to diagnose hepatic damage. Using a rat model and proteomics, the authors conducted further in-depth analysis of the nature of EVs isolated after treatment with a sub-toxic dose of APAP, prior to the manifestation of hepatocellular injury. The results highlighted that most of the secreted EVs isolated from the treated animals possessed different protein composition compared to the non-treated ones in terms of the hepatocytes’ cytoplasmic and cytosolic proteins; moreover, many of the EVs also consisted of matrix proteins secreted by non-parenchymal cells, such as Kupffer, stellate, and endothelial cells. In particular, the authors highlighted that the proteins involved in oxidoreductase activities, as well as those involved in lipid and cholesterol metabolic processes, were present in much smaller quantities within the EVs isolated after treatment than in normal conditions. Furthermore, glycoprotein fibronectin (one of the extracellular matrix components) and Na^+^/K^+^-transporting ATPase (an integral membrane protein of hepatocytes involved in the transport of bile acids across the canalicular membranes) were detected in very high concentrations in EVs that were shed by the liver after APAP treatment. Of particular interest is the authors’ observation that a few members of the cytochrome P450 and glucuronosyltransferase superfamilies, both directly involved in the metabolism of the drug, were not detected or found at very low concentrations in EVs isolated after APAP treatment; this represents key data that could indicate EVs as a new biomarker for the preventive diagnosis of severe oxidative stress, on which APAP hepatotoxicity largely depends [4].

Anti-inflammatory drugs are another example of widely used drugs that are often associated with DILI. Schmidt and colleagues [6] investigated the hepatotoxic properties of the methotrexate (MTX), a drug used for the treatment of rheumatoid arthritis (RA). Indeed, little is known about the precise mechanisms of MTX-induced oxidative stress in hepatocytes and how it leads to the activation of HSCs responsible for liver fibrosis. Using the human hepatic cell line HepaRG, which is known to closely mimic the metabolic activity and gene expression of primary human hepatocytes, and the immortalized cell line hTERT-HSC as surrogates for hepatic stellate cells (HSCs), the authors showed that MTX causes major oxidative stress, which impairs mitochondrial activity by directly affecting the cellular respiration chain; by impairing the production of glutathione, a molecule that acts as a scavenger of ROS (reactive oxygen species); and by triggering the activation of HSCs. This can induce, in a concentration-dependent matter, an increased risk of hepatic necrosis via oxidative stress and liver fibrosis onset. Once again, these results are particularly thought-provoking because they lay the foundations for a very interesting discussion on the role of MTX in the onset of fibrosis in humans. Indeed, recent studies argue that methotrexate may not be a direct cause of liver injury; rather hepatotoxicity may be mediated by other mechanisms [7]. Recent widespread use of the latest 3D human model, organoids, could provide new information on the subject in the future.

The sympathomimetic drug methamphetamine (METH) is one of the most abused neuro-stimulant agents world-wide. Well-known for its neurotoxicity, METH abuse often results in severe hepatotoxicity and cardiovascular system abnormalities. Many of its hepatic side-effects have been described, such as hyperthermia and hyperammonemia, as well as disruption of the CYP1A2 metabolic pathway and the inhibition of cell division. Wang et al. [8] determined, using a mouse model, that the Toll-like receptor 4 (TLR4) signaling pathway is also involved in METH-induced DILI through the activation of several inflammatory pathways. Among these, TLR4-mediated inflammation induced by lipopolysaccharides (LPS) has proven to be of particular interest. Indeed, the overproduction and translocation of LPS form the intestine to the liver that is observed after METH treatment resulted in activation the TLR4 pathway, leading to an increased inflammatory response and hepatotoxicity. As the authors suggest, further investigation must be conducted on this matter as different metabolites might be relevant to METH-induced hepatotoxicity.

One more major cause of acute liver failure caused by xenobiotics is intoxication by mushrooms. Kim and colleagues [9] used a (phospho)proteomic approach to identify the initial signaling pathway involved in hepatotoxicity that occurs after exposure to alpha-amanitin (a-AMA, from the Amanita phalloides), which causes extensive damage by impairing liver and kidney function within a few hours of exposure. The well-established cell-line Huh-7 was treated with a-AMA to induce hepatotoxicity, and global protein phosphorylation changes up to 12h after treatment were profiled based on comparative phosphoproteomic analysis. Between most of the changes observed, the RAS/RAF/ERK signaling cascade was identified as playing a key role in cytotoxicity induced by the molecule. Indeed, in a time-dependent manner, a-AMA exposure resulted in increased activation of the RAS/RAF/ERK signaling pathway, which is involved in aberrant splicing events that lead to cell death. In this context, factors such as U2AF65 and SPF45 also appeared to be involved. Additionally, the authors observed that the damage caused by a-AMA was reduced when ERK inhibitors were used as a countermeasure in the cell culture. Although more validation must still be obtained through in vitro and in-animal models, these observations suggest that ERK 1/2 inhibitor could represent a new therapeutic target to reduce the risk of liver failure caused by the ingestion of *Amanita phalloides*.

Last, but not of least importance, are the two reviews in this Special Issue. Donato and colleagues [10] discussed DILI cases, albeit small, that induce chronic hepatotoxicity after the use of a medications for several days, months, or even years. These cases are likely affected by multiple factors related to a patient’s genetic background, underlying diseases, and associated medications, as well as the pharmacology of the drug. Thus, the authors focused on unfolding existing in vitro models for studying DILI-induced chronic liver failure, from the traditional 2D culture system to organoid technology, as well as emerging technological methods for evaluating chronic DILI, such as microfluidic devices and organ-on-chip models, which are now considered new tools for pharmaceutical and toxicological applications. Moreover, some drugs known to produce chronic hepatotoxicity, the major well-known mechanisms implicated in DILI, and the phenotypic subtypes of chronic DILI (autoimmune-like DILI, steatosis, steatohepatitis, cholestasis, vanishing bile duct syndrome, sinusoidal obstruction syndrome, etc.) are also presented in detail.

Finally, Rausch and colleagues [11] offered a complete description of the role of the farnesoid X receptor (FXR) as a regulator of hepatotoxicity, as it is directly involved in lipid homeostasis and in bile acid (BA) metabolism. The authors fully recapitulate how, under physiological conditions, BAs produced by hepatocytes activate FXR which, being a transcription factor, regulates the encoding of proteins involved in BA synthesis, transport, and degradation, in order to maintain sub-toxic intracellular BA levels. The reduced expression and/or function of FXR leads to increased BA synthesis and reduced BA efflux and degradation, determining the onset of cholestasis. Lipid-induced toxicity is also a condition that is controlled by this receptor; indeed, although the mechanisms by which FXR is involved in insulin resistance and obesity are not easy explain, evidence from animal model studies suggests that the loss of hepatic FXR is at least sufficient to induce the onset of non-alcoholic fatty liver disease (NAFLD) and non-alcoholic steatohepatitis (NASH). Furthermore, the farnesoid X receptor family also seems to play a role in protecting the liver from some forms of DILI, such as APAP and amoxicillin/clavulanate hepatotoxicity, as well as drug-induced cholestasis. However, as the abovementioned authors underline in their work, the myriad of signaling pathways modulated by FXR, the existence of several isoforms in humans, and its specific expression across several tissues (especially the intestine) make it difficult to identify FXR as a real therapeutic target.
